# Assessment of selection criteria using multi-year study for effective breeding program of* Zingiber officinale* L

**DOI:** 10.7717/peerj.15966

**Published:** 2023-09-01

**Authors:** Twahira Begum, Roktim Gogoi, Ankita Gogoi, Tanmita Gupta, Sanjoy Kumar Chanda, Himangshu Lekhak, Mohan Lal

**Affiliations:** Agrotechnology and Rural Development Division, CSIR-North East Institute of Science and Technology, Jorhat, Assam, India

**Keywords:** ANOVA, Genotypic coefficient of variation, Phenotypic coefficient of variation, Heritability, Correlation, Path analysis

## Abstract

**Background:**

Ginger has been an important cash crop with numerous applications since ancient times. As the demand for ginger is ever-growing and being a seasonal crop, a high-yielding variety of ginger would be economically profitable.

**Methods:**

In this study, 150 germplasm were collected from different regions of NE India and evaluated for three years in CRBD design with three replications. The present study thus focused on the variability, association, and diversity studies for the first time on 150 ginger germplasm from across North East India. The genotypic and phenotypic coefficient of variation, heritability, correlation, and path analysis were evaluated for the germplasm.

**Results:**

Analysis of variance (ANOVA) revealed considerable differences among the studied germplasm for studied characters, revealing sufficient variability in the materials. The Mahalanobis D^2^ and Tocher methods grouped the 150 ginger germplasm into ten clusters. Based on the results of the path coefficient analysis determined for essential oil yield and rhizome yield per plant, it can be concluded that the characters’ initial rhizome weight, the weight of mother rhizome, and weight of secondary rhizome were the most important and appeared promising in improving the overall yield potential of ginger rhizome and essential oil yield. Thus, selection based on the identified traits would lead to an effective ginger breeding program for higher rhizome and essential oil yield.

## Introduction

*Zingiber officinale* Rosc. or ‘Ginger’ in English belongs to the Zingiberaceae family and is a herbaceous perennial plant commonly diploid with a chromosome number of 2*n* = 22. Ginger is one of the oldest spices known and used by mankind and it is mostly used as fresh (Begum et al., 2018). The most valuable part of the plant is the rhizome. It has been used in Ayurveda, as a spice in daily life and a natural remedy for colds, coughs, and other ailments since ancient times (Begum et al., 2022). The different chemical components of ginger are accountable for its various valuable pharmacological properties.

Ginger is widely cultivated worldwide in many regions, such as China, India, West Indies islands, Australia, Indonesia, Nigeria, and Japan (Sivaprasad & Balachandran, 2022; Abubacker, 2009). In India, it is mainly grown in Assam, Arunachal Pradesh, Kerala, Meghalaya, Nagaland, Mizoram, Tripura, Manipur, Orissa, Madhya Pradesh, Karnataka, and Sikkim (Srinivasan et al., 2008; Rahman et al., 2009). In Northeast India, the highest ginger productivity was recorded in Mizoram, Assam, Arunachal Pradesh, and Nagaland (Rahman et al., 2009; Begum et al., 2018). The cultivated ginger in the North East region of India is reported to have the maximum variability (Begum et al., 2022). Northeast India is the country’s largest (72%) ginger producer (Rahman et al., 2009).

Ginger is an important cash crop among all spices, has wide usage worldwide, and has wide application as an ingredient in beverages and foods (Begum et al., 2020; Gupta, 2008). The worldwide consumption of ginger is increasing day by day. Ginger is widely used as a source of raw materials for various therapeutic and flavoring industries throughout the world (Munda et al., 2018). Ginger’s characteristic pungency and piquant flavor have led to extensive consumption as a spice and wide application in beverages, foods as a preserve in sugar syrup (murabba), carbonated drinks and liquors (Spices Board of India, 2022).

Among all spices, ginger is the major cash crop supporting income and improving the socio-economic status of ginger cultivators. Ginger’s characteristic pungency and flavor are due to the oleoresins and essential oil, which are the highly valued products (Begum et al., 2018). Due to its distinct aroma, flavor, and pungency, essential oil and oleoresins are extensively used in cosmetic industries, perfumeries and flavor (Singh et al., 2008). Ginger is mostly used in fresh form, and in addition to that, dried ginger powder is also used in the manufacturing of ginger brandy, beer, and wine (Yadav et al., 2004). Due to the seasonal availability of fresh ginger, it is usually dried and stored for further use and is known as dry ginger. The ginger that gives high biomass after drying is called high-dry ginger (Baruah et al., 2019).

The genetic variability for agronomic traits is the breeding program’s key component for broadening the gene pool. For a successful selection process in breeding, the genetic coefficient of variation (GCV) and the heritability estimate give a fair estimate for the expected amount of advance from the selection. The amount of genetic variability is the determining factor for the genetic advance for selection. The various agronomic traits are interlinked with other agronomic traits. Thus, the study of the correlation of the traits is of significance. In the crop improvement program, yield is a prime objective in the breeding program. However, yield is complex and is regulated by the combination of many other characteristics. Therefore, insight into the direct and indirect effects of the various attributes on crop yield is of paramount importance. In this regard, path analysis is essential to analyze the multiple attributes. In plant breeding programs, the correlation study and path analysis gives improved information into the relationship of cause and effect among the agronomical traits.

Although very little work on the morphological diversity of ginger germplasm has been done, still various studies have been conducted on ginger cultivars of different regions. Being a biodiversity hotspot, Northeast India is a rich hub of ginger diversity. The biodiversity of ginger from the entire northeast region has not been exploited so far, which could lead to promising lines of ginger for high rhizome and essential oil yields. Since the demand for ginger is ever-growing and is a seasonal crop, a good variety of ginger with a better shelf-life would be economically more profitable. Given the previous reports, only limited germplasm has been studied, which do not account for stable, reliable data. Hence, the present study focuses on studying variability parameters for the first time on 150 ginger germplasm across North east India. The study would be of great benefit to the ginger breeding program.

## Materials & Methods

### Collection of ginger germplasm

In total, 150 ginger germplasm were collected across Northeast India. The collection sites were Assam, Meghalaya, Arunachal Pradesh, Mizoram, Manipur, Nagaland, and Sikkim. All the collected germplasm was identified by the plant breeder of CSIR-NEIST, Jorhat and maintained at the experimental farm of CSIR-NEIST, Jorhat, Assam. The herbarium specimen was submitted to the herbarium record of the department.

### Experiment layout

The 150 ginger germplasm were planted in a 2 × 2 m plot size with triplicates in RBD (Randomized Block Design) at the experimental farm of CSIR-NEIST, Jorhat. The plant to plant and row to row distance of 35 × 35 cm was maintained. The experiment was conducted for three years (spring 2018, 2019 and 2020), respectively.

### Morphological data recording

The detailed data recording was carried out by considering sixteen agronomical traits, including plant height (PH) (cm), number of tillers per plant (NTP), number of leaves per plant (NLP), leaf length (LL) (cm), leaf width (LW) (cm), number of mother rhizome (NMR), number of primary rhizomes (NPR), number of secondary rhizomes (NSR), initial rhizome weight (IRW) (g), the weight of mother rhizome (WMR) (g), the weight of primary rhizome (WPR) (g), the weight of secondary rhizome (WSR) (g), the diameter of mother rhizome (DMR) (cm), diameter of primary rhizome (DPR) (cm), diameter of secondary rhizome (DSR) (cm) and rhizome yield per plant (RYP) (t/ha). The morphological data were recorded from five randomly selected plants from each replication for each germplasm. In addition to that, the essential oil yield (EOY) (%) was also recorded. As for the essential oil yield, 300 g of shade-dried rhizome were hydrodistilled using Clevenger apparatus for 8 $ \frac{1}{2} $ h, after which the isolated essential oil was collected and measured, and the moisture content was removed by treatment with anhydrous sodium sulphate. Essential oil isolation was also carried out in triplicates.

### Statistical analysis

The average morphological data of three years were used for the present study. Mahalanobis D^2^ analysis was performed using Indostat software (8.2 version; https://www.indostat.org/) for the genetic diversity study. The Tocher method was used for the cluster analysis and the inter and intra distance of the clusters were found using Mahalanobis Euclidean Distances. The Analysis of variance (ANOVA), genetic advance (GA), genetic gain (GG) (Johnson, Robinson & Comstock, 1955a; Johnson, Robinson & Comstock, 1955b), and heritability in broad sense (HBS) (Johnson, Robinson & Comstock, 1955a; Johnson, Robinson & Comstock, 1955b; Burton & Devane, 1953) were analyzed along with the variability parameters genotypic coefficient of variance (GCV) and phenotypic coefficient of variance (PCV) (Al-Jibouri, Miller & Robinson, 1958) for the morphological characters. Association studies like path and correlation coefficient analysis (Dewey & Lu, 1959) were performed to determine the direct and indirect effects of different parameters on RYP and EOY.

## Results

The studied 150 germplasm of ginger were from across seven states of Northeast India. The morphological data for the 150 germplasm of ginger were recorded for three consecutive seasons spring 2018, 2019 and 2020. Pooled data from three years was used to estimate variability parameters, correlations, path, and morphological diversity.

The ANOVA was analyzed for three-year pooled data (Table 1). ANOVA revealed considerable differences among the studied germplasm for different characters revealing sufficient variability in the materials. The highest value of GCV and PCV was observed for WMR (42.126, 50.802) followed by NTP (21.947, 96.481), indicating high character diversity. While moderate GCV and PCV were recorded for RYP (28.542, 32.631) followed by EOY (27.017, 28.348) and WSR (23.949, 43.282). For all the characters studied, the PCV was found to be higher than that of GCV.

The highest difference between PCV and GCV was observed for NTP (21.947, 96.481), followed by IRW (10.346, 35.519) and WSR (23.949, 43.282), indicating the influence of environmental effects. While the lowest difference between GCV and PCV was observed in EOY (27.017, 28.348) followed by LL (11.794, 15.685), RYP (28.542, 32.631), DMR (7.335, 11.565), PH (9.601, 14.35) and DPR (6.115, 11.493) (Table 1). High heritability coupled with high GA was observed for EOY (90.8%, 0.810), followed by RYP (76.5%, 0.75). Moderate heritability was observed for WMR (68.8), LL (56.5), NTP (50.2), PH (44.8), and DMR (40.2). While low heritability was observed for IRW (10.1), WSR (10.2) and DSR (14.3). Moderate heritability with moderate genetic advance was observed for LL (56.5, 3.118), followed by NTP (50.2, 1.924), PH (44.8, 7.131), and DMR (40.2, 0.816). Meanwhile, low heritability coupled with low genetic advance was observed for IRW (10.1, 0.001), followed by WSR (10.2, 0.001), and DSR (14.3, 0.210).

**Figure 1:**
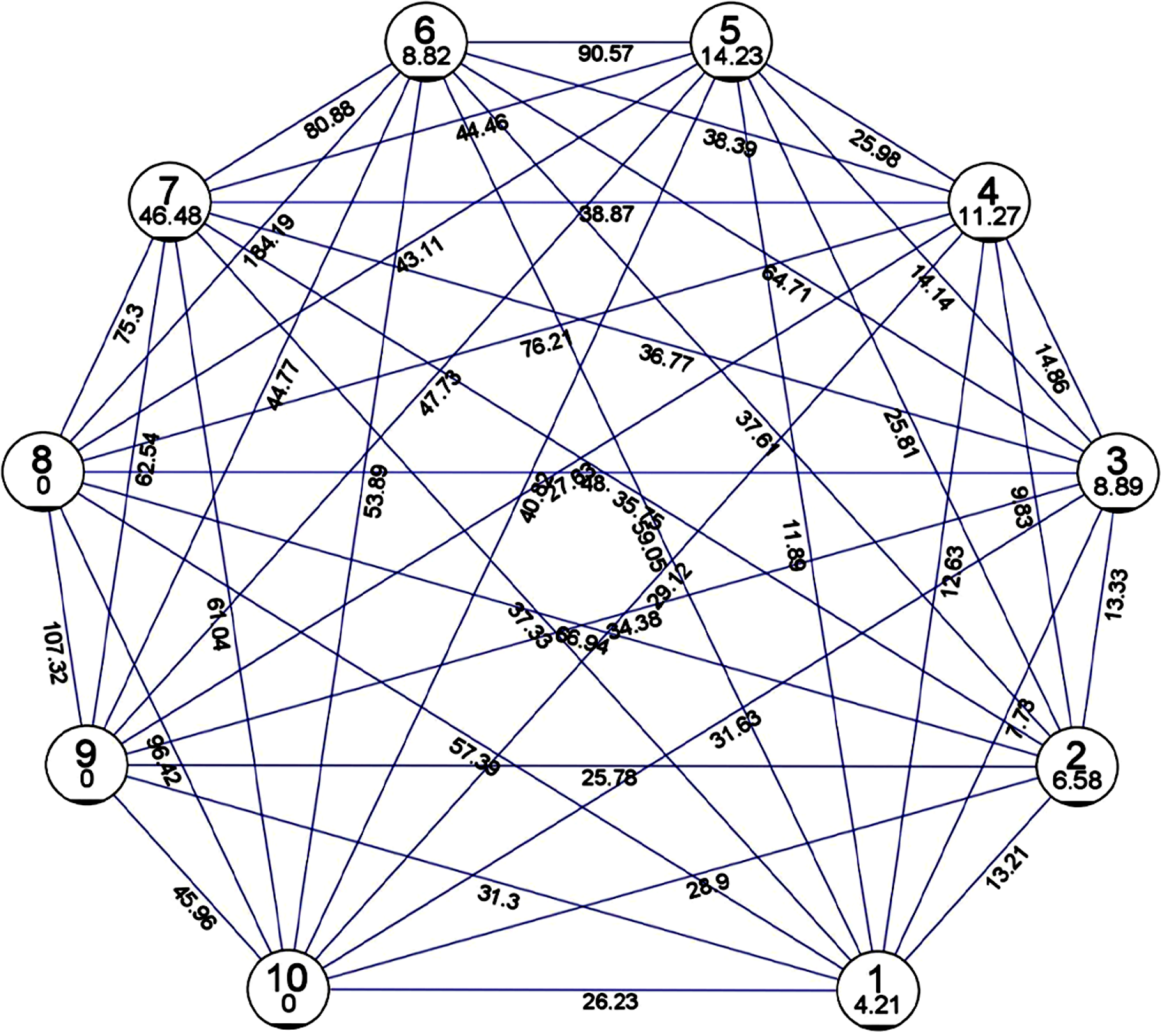
Mean inter and intra cluster distance by Euclidean method and Tocher method. Mean inter and intra cluster distance among genotypes of *Zingiber officinale* using D^2^ statistics by Euclidean method and Tocher method (not to scale).

The Mahalanobis D^2^ method was used to analyze the genetic divergence based on their morphological data. The ginger germplasm was grouped into ten (10) clusters based on the traits under study. The maximum intra-cluster distance as per Mahalanobis Euclidean Distance was 46.48 and the minimum was 0 (Fig. 1). Cluster 7 (46.48) was found to have the maximum intra-cluster distance followed by cluster 5 (14.23), cluster 4 (11.27), cluster 3 (8.89), cluster 6 (8.82), cluster 2 (6.58) and cluster 1 (4.21). While minimum intra-cluster distance (0) was found in cluster 8, cluster 9 and cluster 10. The germplasm belonging to clusters 8 and 6 revealed maximum divergence, with 184.19 as the inter-cluster distance while clusters 1 and 3 exhibited minimum divergence which had inter-cluster distance of 7.73.

**Table 1:** Analysis of variance for the morphological characters of *Zingiber officinale* for pooled data (2018, 2019, 2020).

Trait	Source of variation and mean squares (ANOVA)			Estimation of genetic parameter				Mean performance		
	Replication (DF = 2)	Genotypes (DF = 149)	Error (DF = 298)	GCV%	PCV%	h^2^(bs) (%)	G.A 5%	Mean	S.E	C.D 5%
Plant height	699.53	113.354**	33.0412	9.601	14.350	44.8	7.131	53.893	4.6933	9.2363
No. of tiller	101.04	359.496**	308.9261	21.947	96.481	50.2	1.924	18.708	14.3510	8.242
No. of leaf	3.256455	9.448*	4.6409	7.152	14.117	25.7	1.321	17.700	1.7590	3.4616
Leaf length	33.779620	15.272*	3.1153	11.794	15.685	56.5	3.118	17.069	1.4411	2.8361
Leaf width	0.168877	0.107*	0.0445	7.411	13.080	32.1	0.169	1.957	0.1722	1.3390
No. M Rh	0.810231	0.477**	0.2501	12.099	25.080	23.3	0.274	2.277	0.4084	0.8037
No. P Rh	0.554097	3.079**	1.3700	16.837	31.066	29.4	0.843	4.483	0.9557	1.8808
No. S Rh	3.843983	3.849**	2.0533	12.747	26.829	22.6	0.757	6.070	1.1700	2.3025
Int. Weight	0.244238	0.305*	0.305206	10.346	35.519	10.1	0.001	0.156	0.4511	0.8877
Weight M Rh	0.000762	0.008*	0.000114	42.126	50.802	68.8	0.027	0.038	0.0087	0.0172
Weight P Rh	0.001389	0.007*	0.000286	23.505	39.432	35.5	0.015	0.053	0.0138	0.0272
Weight S Rh	0.291516	0.302*	0.302161	23.949	43.282	10.2	0.001	0.065	0.4488	0.8833
Dia M Rh	0.548774	1.749*	0.579424	7.335	11.565	40.2	0.816	8.513	0.6215	1.2231
Dia P Rh	0.489540	1.119*	0.512579	6.115	11.493	28.3	0.493	7.357	0.5846	1.1504
Dia S Rh	0.121738	0.651**	0.433658	4.681	12.370	14.3	0.210	5.751	0.5377	1.0581
Rhizome yield (t/ha)	0.001527	0.006**	0.000534	28.542	32.631	76.5	0.75	14.6	0.0189	0.0371
EO %	0.011468	0.009**	0.000320	27.017	28.348	90.8	0.810	0.208	0.0146	0.0287

**Notes.**

DF degree of freedom M mother Rh rhizome P primary S secondary GCV genotypic coefficient of variation PCV phenotypic coefficient of variation Int weight initial weight of single rhizome Dia diameter EO essential oil GCV genotypic coefficient of variation PCV phenotypic coefficient of variation h^2^ (bs) heritability in broad sense GA genetic advance

**Figure 2:**
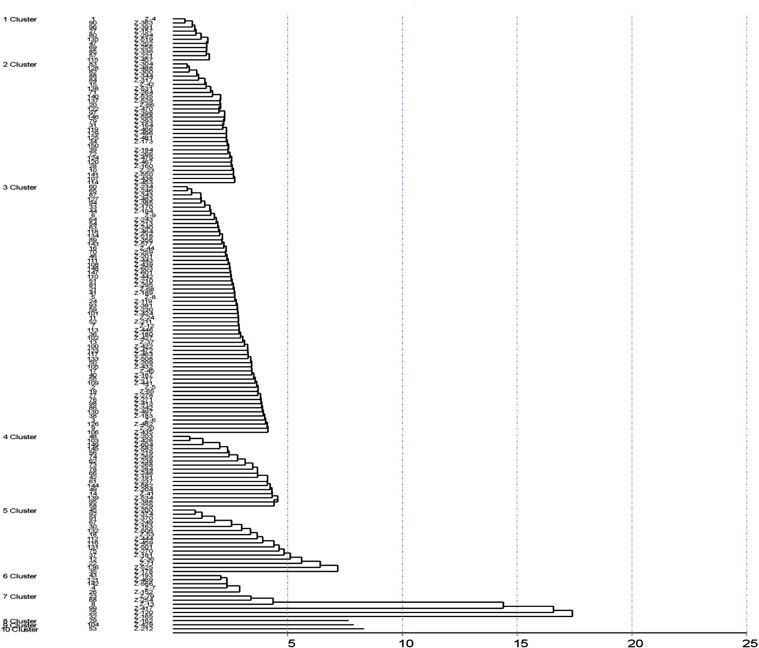
Cluster analysis by Tocher method.

The Tocher method was used for preparing the dendrogram of the 150 accessions of ginger in which 10 clusters were formed (Fig. 2). Cluster 3 has the highest of 61 germplasm while clusters 8, 9, 10 had a single cluster indicating unique accession. Cluster 1 was found to consist of 11 germplasm. Cluster 2 constituted of 30 germplasm, cluster 3 of 61 germplasm, cluster 4 of 18 germplasm, cluster consisted five of 16 germplasm, cluster 6 included five germplasm and cluster 7 consisted of six germplasm.

The genotypic correlation matrix for the seventeen morphological characters has been presented in Table 2. The PH was found to be significantly and positively correlated to NTP. While PH was non-significantly but positively correlated to NLP, DPR and DSR. Meanwhile, the character PH was found to be correlated negatively with IRW, WSR and EOY. The character NTP was found to be negatively and significantly correlated to RYP.

**Table 2:** Genotypic correlation matrix for the morphological characters of *Zingiber officinale* for pooled data (2018, 2019, 2020).

	PH	NTP	NLP	LL	LW	NMR	NPR	NSR	IRW	WMR	WPR	WSR	DMR	DPR	DSR	EOY	RYP
PH	1.0000																
NTP	0.5145^*^	1.0000															
NLP	0.3693	0.5097^**^	1.0000														
LL	0.1745	−0.0062	0.2575	1.0000													
LW	0.1017	0.3400	0.0890	0.2112	1.0000												
NMR	0.1527	0.1341	0.1781	0.0343	0.0759	1.0000											
NPR	0.0974	0.1539	−0.0236	−0.1988	−0.2353	0.1651	1.0000										
NSR	0.0661	−0.5125	−0.2873	−0.1397	0.0050	0.3251	0.3005	1.0000									
IRW	−0.5385^**^	−0.8594	−0.1825	0.1168	0.4037	−0.6192	−0.8895	−0.1510	1.0000								
WMR	−0.0603	−0.2093	−0.1693	−0.1090	0.0339	0.0332	−0.0893	0.0775	−0.1440	1.0000							
WPR	0.2981	−0.3133	0.1512	0.0066	−0.0414	−0.2512	0.1713	0.2871^**^	0.2528	0.0151	1.0000						
WSR	−0.8417	−0.4613	−0.2779	0.2250	0.4145	−0.5805	−0.0004	−0.6127	0.1858	−0.1728	0.4823	1.0000					
DMR	−0.0207	0.2959	−0.2635	0.0060	0.1374	−0.3536	−0.0686	0.0195	0.9182	−0.0904	0.1567	0.9829^**^	1.0000				
DPR	0.3303	0.1339	0.0584	0.0502	0.3226	0.1051	−0.0666	0.0114	0.2845	0.1307	0.1940	0.0057	0.3623	1.0000			
DSR	0.3987	0.2253	0.0540	0.0123	0.3007	−0.3321	0.2246	0.0110	0.3527	−0.0642	0.3608	0.1420	0.7585	0.6587	1.0000		
EOY	−0.1334	0.2296	0.0224	−0.2034	−0.1333	−0.0782	0.1394	−0.1546	0.6630^**^	0.1825	0.1306	0.6534^**^	−0.1821	−0.3222^**^	−0.3523^**^	1.0000	
RYP	0.0346	−0.2461^**^	−0.0577	0.0678	−0.0360	0.0498	0.0641	0.1454^**^	0.0718^*^	0.0008	0.0573^*^	0.0279	0.0846^*^	0.0422	0.0870^*^	−0.3407^***^	1.0000

**Notes.**

* Significant at 5%.

** Significant at 1% level.

For the trait rhizome yield, the economically important character was found to be correlated significantly and positively with NSR, IRW, WPR, DMR and DSR. However, the RYP was found to be correlated negatively and significantly with EOY and NTP. It was found to be correlated positively but non-significantly to PH, LL, NMR, NPR, WMR, WSR, and DPR. Meanwhile, rhizome yield was found to be correlated negatively and non-significantly with NLP and LW.

For the EOY character, it was found to be correlated negatively and significantly with DPR and DSR. The EOY was found to be correlated significantly and positively with IRW and WSR. However, the EOY was found to be correlated negatively and non-significantly with PH, LL, LW, NMR, NSR, and DMR. While the EOY was found to be correlated positively and non-significantly with the traits like NTP, NLP, NPR, WMR and WPR.

The path coefficient was analyzed for EOY and RYP using 17 morphological traits. The matrix of the path coefficient for EOY is presented in Table 3. The path analysis revealed that the maximum direct effect was exhibited by IRW (0.9851) followed by WMR (0.7913) and WSR (0.572) on EOY. The IRW was found to correlate with EOY, which was found to be significant and positive mainly due to its direct and indirect effects via DPR. The WMR was found to have a significant and positive correlation with EOY mainly due to its direct effect and an indirect effect via WSR and LL. The WSR was found to be correlated positively and significantly with EOY mainly due to its direct effect and an indirect effect via PH, NLP, and NSR. Furthermore, the path coefficient analysis matrix revealed that RYP (−0.2362) has a direct negative correlation with EOY followed by NSR (−0.2028) and LL (−0.192).

**Table 3:** Path coefficient analysis of pooled data (2018, 2019, 2020) for essential oil yield showing direct and indirect effects of different characteristics.

	PH	NTP	NLP	LL	LW	NMR	NPR	NSR	IRW	WMR	WPR	WSR	DMR	DPR	DSR	RYP	EOY
																	r
PH	**0.0628**	0.0323	0.0232	0.011	0.0064	0.0096	0.0061	0.0041	−0.1091	−0.0038	0.0187	−0.2413	−0.0013	0.0207	0.025	0.0022	−0.1334
NTP	0.1225	**0.3158**	0.161	−0.002	0.1074	0.0424	0.0486	−0.1619	−0.5873	−0.0661	−0.099	0.219	0.0935	0.0423	0.0711	−0.0777	0.2296
NLP	0.0383	0.0528	**0.1037**	0.0267	0.0092	0.0185	−0.0024	−0.0298	0.2789	−0.0176	0.0157	−0.45	−0.0273	0.0061	0.0056	−0.006	0.0224
LL	−0.0135	0.0012	−0.0196	**−0.192**	−0.0406	−0.0066	0.0482	0.0368	−0.0224	0.0209	−0.0013	−0.0232	−0.0011	−0.0096	0.0324	−0.013	−0.2034
LW	0.0098	0.0126	0.0085	−0.0452	**0.0658**	0.0073	−0.0626	−0.0405	0.0187	0.0045	−0.004	−0.0497	0.0032	0.0205	−0.0688	−0.0134	−0.1333
NMR	−0.0511	−0.0448	−0.0595	−0.0115	−0.0254	**−0.1342**	−0.0552	−0.1087	0.0596	−0.0189	0.084	0.0767	0.1182	−0.0351	0.111	0.0167	−0.0782
NPR	0.0513	0.0811	−0.0124	−0.1047	−0.124	0.087	**0.5268**	0.1583	−0.6343	−0.047	0.0902	0.0538	−0.0361	−0.0351	0.1183	−0.0338	0.1394
NSR	−0.1138	−0.1767	0.0598	0.0291	−0.0487	−0.0677	−0.0626	**−0.2082**	0.5688	−0.0161	−0.0598	0.0504	−0.0441	−0.0324	−0.0023	−0.0303	−0.1546
IRW	−0.1108	−0.6902	−0.5148	0.0629	0.0448	−0.9166	−0.7379	0.4185	**0.9851**	−0.0086	0.5856	0.4644	0.5287	0.898	0.2702	−0.6163	0.663
WMR	0.0481	0.113	−0.7552	0.7048	−0.1518	−0.1277	0.0351	−0.634	0.8925	**0.7913**	−0.5133	0.5588	0.0726	−0.6416	−0.1826	−0.0275	0.1825
WPR	0.8926	0.2939	−0.0024	−0.175	0.0972	0.6505	−0.5339	−0.5999	−0.1641	−0.3998	**0.4726**	−0.7674	−0.1473	0.1356	−0.1393	0.5173	0.1306
WSR	0.9747	0.0578	0.6175	−0.625	−0.9164	0.2035	0.1564	0.858	−0.2378	0.3744	−0.3514	**0.572**	−0.1432	−0.2103	−0.7569	0.0801	0.6534
DMR	0.0027	0.039	−0.0347	0.0008	0.0181	−0.0466	−0.009	0.0026	−0.2257	−0.0119	0.0206	−0.1331	**0.1318**	−0.0477	0.0999	0.0111	−0.1821
DPR	0.073	0.0296	0.0129	0.0111	−0.0713	0.0232	−0.2147	0.0025	−0.2628	0.0289	−0.4116	0.0013	0.08	**0.2209**	0.1455	0.0093	−0.3222
DSR	0.4412	−0.2493	−0.0597	−0.0136	−0.2018	0.3675	−0.2486	−0.0122	−0.3903	0.0711	−0.2994	−0.1572	0.8395	−0.429	**−0.1068**	0.0963	−0.3523
RYP	−0.0082	0.0581	0.0136	−0.016	0.0085	0.0118	0.0151	−0.0343	−0.4728	−0.0002	−0.1495	0.4789	−0.02	−0.01	0.0205	**−0.2362**	−0.3407

**Notes.**

* Significant at 5%.

** Significant at 1% level, Bold values indicate direct effects.

The matrix of path coefficient analysis for RYP is presented in Table 4. It revealed that the IRW (0.6139) exhibited maximum direct effect followed by WMR (0.4681) and WSR (0.413). The IRW was correlated significantly and positively with RYP mainly due to its direct and indirect effects via EOY and WSR. The WMR was correlated significantly and positively with EOY mainly due to its direct and indirect effects via IRW. The WSR was correlated significantly and positively with EOY mainly due to its direct and indirect effects via NTP, WMR and DSR. Meanwhile, the RYP was found to be negatively correlated with EOY (−0.5539) followed by NMR (−0.3472), DSR (−0.3381) and DMR (−0.3157).

**Table 4:** Path coefficient analysis of pooled data (2018, 2019, 2020) for rhizome yield showing direct and indirect effects of different characteristics.

	PH	NTP	NLP	LL	LW	NMR	NPR	NSR	IRW	WMR	WPR	WSR	DMR	DPR	DSR	EOY	RYP
PH	**−0.0891**	−0.0459	−0.0329	−0.0156	−0.0091	−0.0136	−0.0087	−0.0059	0.2053	0.0054	−0.0266	0.1225	0.0018	−0.0294	−0.0355	0.0119	0.0346
NTP	0.2021	**0.3929**	0.2003	−0.0024	0.1336	0.0527	0.0605	−0.2014	−0.7306	−0.0822	−0.1231	−0.4961	0.1163	0.0526	0.0885	0.0902	−0.2461
NLP	0.0171	0.0236	**0.0463**	0.0119	0.0141	0.0083	−0.0067	−0.0133	−0.0939	−0.0078	0.007	−0.0583	−0.0122	0.0027	0.0025	0.001	−0.0577
LL	0.0048	−0.0002	0.0072	**0.0278**	0.0059	0.001	−0.0055	−0.0039	0.0099	−0.003	0.0002	0.0163	0.0018	0.0014	0.0098	−0.0057	0.0678
LW	−0.0211	0.0704	−0.0184	0.0438	**−0.2072**	−0.0157	0.0488	−0.001	0.0751	−0.007	0.0086	−0.0159	−0.0285	0.0668	−0.0623	0.0276	−0.036
NMR	−0.053	−0.0466	−0.0618	−0.0119	−0.0264	**−0.3472**	−0.0573	−0.1129	0.2568	−0.0115	0.0872	0.2433	0.1228	−0.0365	0.0776	0.0272	0.0498
NPR	0.0124	0.0195	−0.003	−0.0743	−0.0299	0.021	**0.1269**	0.0381	−0.2399	−0.0113	0.0217	0.1539	−0.0087	−0.0085	0.0285	0.0177	0.0641
NSR	0.0136	−0.1055	−0.0591	−0.0288	0.001	0.0669	0.0618	**0.2058**	−0.0343	0.0175	0.0591	−0.0294	0.004	0.0023	0.0023	−0.0318	0.1454
IRW	−0.006	−0.1447	0.0313	−0.7195	0.3146	−0.2659	−0.3618	0.7579	**0.6139**	−0.818	0.6294	0.5091	−0.9367	0.0976	−0.2531	0.6237	0.0718
WMR	0.3785	0.2605	0.6838	0.3025	−0.3377	−0.3097	−0.5248	−0.0589	0.6819	**0.4681**	−0.5961	0.2895	−0.5683	−0.1582	0.2058	−0.7161	0.0008
WPR	0.2749	0.7466	−0.6534	−0.2057	0.2776	0.8153	−0.328	0.9309	−0.3726	−0.4698	**0.1091**	0.0035	−0.8737	−0.035	−0.2257	0.0633	0.0573
WSR	−0.8391	0.9939	−0.8506	−0.7773	−0.1677	0.649	0.0203	0.9095	−0.2469	0.744	−0.8107	**0.413**	−0.3435	−0.2211	0.0719	0.4832	0.0279
DMR	0.0066	−0.0934	0.0832	−0.0019	−0.0434	0.1116	0.0217	−0.0062	0.9213	0.0285	−0.0495	−0.2819	**−0.3157**	−0.1144	−0.2394	0.0575	0.0846
DPR	0.066	−0.0268	0.0117	0.01	−0.0645	0.021	−0.0133	0.0023	−0.0569	0.0261	0.0388	0.0011	−0.1924	**0.1998**	0.0837	−0.0644	0.0422
DSR	0.1348	−0.0761	−0.0182	−0.0042	−0.0617	0.1123	−0.0259	−0.0037	0.1192	0.0558	−0.122	−0.048	0.2664	−0.0227	**−0.3381**	0.1191	0.087
EOY	0.0739	−0.0572	−0.0124	0.1926	0.0738	0.0933	−0.0772	0.0856	−0.2376	0.0381	−0.0723	−0.3619	0.1009	0.1785	0.1951	**−0.5539**	−0.3407

**Notes.**

* Significant at 5%.

** Significant at 1% level, Bold values indicate direct effects.

## Discussion

For a successful breeding program, the presence of genetic variability in a crop is a determinant. High variance among the crops enhances the probability of the evolvement of crops possessing elite traits. The genotypic facts are inferred from phenotype data which are the outcome of the genotype and environment interaction. Since the environment dramatically influences many qualitative and quantitative traits, estimating parameters like GCV, HBS, and GG would be helpful for categorizing the traits under heritable and non-heritable components. Such an approach would help the breeder develop and formulate an effective selection program targeted for crop improvement.

The ANOVA revealed significant differences among the genotypes for various characters, indicating sufficient variability was present among the studied material. In the present study, the ANOVA revealed GCV was lower than PCV for all the characters. While the lowest difference between GCV and PCV was observed in EOY followed by LL, RYP, DMR, PH, DPR, LW, and NLP indicating that the variability was primarily due to genotypic difference. While the high difference between GCV and PCV was observed for the traits NTP, NMR, NPR, NSR, IRW, WMR, WPR, WSR, and SDR indicating the influence of environmental effects. Hence selection of such characters should be performed carefully considering environmental factors. A previous study reported that estimated variability parameters for different characters revealed high mean values for most studied characters (Jatoi & Watanabe, 2016). Another study evaluated 25 ginger genotypes and observed significant variation for different characteristics like PH, plant girth, DM, length, diameter, and number of the primary rhizome and RYP (Ravishanker et al., 2014).

High GCV and PCV values were recorded for WMR, followed by NTP, indicating high character diversity. Meanwhile, moderate GCV and PCV were reported for RYP, followed by EOY and WSR. On the other hand, low GCV and PCV were seen for DMR, DPR, DSR, NLP, and LW, indicating that environmental fluctuations highly influence these traits.

Estimates of PCV and GCV do not solitarily assess the amount of heritable variations for which further heritability estimation is done. High heritability (>75%) was observed for EOY and RYP, while moderate heritability (>50%) was recorded for WMR, LL and NTP. This indicated a high transmission index for the characters. It has been reported that GCV, together with heritability would provide a clear idea of the efficiency of selection as GCV depicts the amount of genetic variation, while the proportion of transmittance of the variability of a character to its progenies is estimated by the heritability (Burton, 2007). However, further reports suggested that heritability and GA would be more effective in forecasting the resultant phenotypic expression effect for the selection (Johnson, Robinson & Comstock, 1955a; Johnson, Robinson & Comstock, 1955b). High heritability and high GA was observed for EOY, followed by RYP. Moderate heritability with moderate GA was observed for LL, NTP, PH, and DMR. Therefore, these characters might be exhibiting a predominance of an additive gene effect. Thus, selecting these traits would be effective for the genetic improvement of RYP and EOY in ginger. Similar results were reported by previous ginger germplasm studies (Jatoi & Watanabe, 2016; Singh et al., 2003; Rao et al., 2004; Baranwal et al., 2012).

A previous study on 13 ginger germplasm for two years reported that based on cluster analysis, was grouped into three clusters. However, the germplasm assignment into the clusters differed for both years. During the first-year cluster analysis, cluster I was grouped based on the genotypes possessing high mean values for the studied traits, while a similar observation was revealed for cluster II during the second year. The clustering pattern was not based on collection sources but was instead found to be based on quantitative characters (Jatoi & Watanabe, 2016). The genetic divergence was analyzed by Mahalanobis D^2^ method based on their morphological data, which grouped the germplasm into ten (10) clusters. According to Mahalanobis Euclidean Distance, 46.48 and 0 were reported as the maximum and minimum intra cluster distances observed. Cluster 7 exhibited the maximum intra-cluster distance while cluster 8, cluster 9 and cluster 10 exhibited the minimum intra-cluster distance (0). The genotypes of cluster 8 and 6 exhibited maximum divergence, and clusters 1 and 3 revealed minimum divergence among them. As per the Tocher method, the ginger germplasm were grouped into 10 clusters among which cluster 3 has the highest of 61 germplasm while clusters 8, 9, 10 had a single cluster indicating unique accession.

The information on the genetic correlation of RYP and EOY is necessary; their components and various quality characteristics are of paramount importance in a breeding program that aims at combining desirable quality and agronomic parameters with high yield potential. Therefore, the association study would provide in-depth data on the nature, direction, and extent of selection. The rhizome yield was positively and significantly correlated with NSR, IRW, WPR, DMR, and DSR, and selection based on these traits would be more rewarding. The EOY was reported to be correlated positively and significantly with IRW and WSR. However, a previous study reported that plant height, leaves per tiller and tiller thickness seemed significant as these traits were found to directly influence the yield, which differs from our report (Jatoi & Watanabe, 2016). A previous study on correlation analysis of ginger genotypes for RYP revealed a positive and significant correlation with NMR per plant, number of finger rhizomes per plant, and NTP (Rajyalakshmi & Umajyothi, 2014). Previous reports also suggested that RYP was positively correlated with NTP, PH, and rhizome thickness (Ravi et al., 2017; Rao et al., 2004; Anargha et al., 2020).

The results of the correlation study do not clarify the contribution factor of each character. Moreover, since association studies include more variables, revelation on the direct association becomes significant and complex. For finding the associated contributing factors of a trait, the path coefficient analysis is of great aid in classifying the indirect and direct causes of association or correlation. It provides an insight into the traits contributing to producing a given correlation (Jain, Elangovan & Patel, 2010). The path coefficient analysis also provides an estimate of the significance of each causal factor, thereby providing an estimate for the distribution of weightage to each contributing trait in determining factors to be considered for the genetic improvement program. The path coefficient analysis for EOY revealed that the IRW displayed maximum direct effect followed by WMR and WSR on EOY. The IRW exhibited a significant and positive correlation on EOY mainly due to its direct and indirect effects via DPR. The WMR exhibited significant and positive correlation on EOY mainly due to its direct effect and an indirect effect via WSR and LL. The WSR exhibited a significant and positive correlation on EOY mainly due to its direct effect and an indirect effect via PH, NLP, and NSR.

The matrix of path coefficient analysis for RYP revealed that the IRW exhibited maximum direct effect followed by WMR and WSR. The IRW was correlated significantly and positively with RYP mainly due to its direct and indirect effects via EOY and WSR. The WMR was found to have a significant and positive correlation on EOY mainly due to its indirect and direct effects via IRW. The WSR was found to have a significant and positive correlation with EOY mainly due to its direct effect and indirect effect via NTP, WMR and DSR. Previous report on ginger revealed that for improving the high yield trait selection should be done based on rhizome, thickness of secondary rhizome, and leaflet number (Abraham & Latha, 2003). Previous findings revealed that for the trait high RYP highest direct positive effect was exerted by the trait NLP, followed by the traits number of shoots and rhizome thickness, respectively, indicating the effectiveness of these characters for improvement in the yield of ginger (Basak et al., 2019). Another study reported on the association analysis for two years in the ginger germplasm, and positive and significant correlations were observed for different quantitative traits. The characters plant height, tiller thickness, and leaves per tiller appeared to be of prime importance as they directly influenced the rhizome weight and rhizome thickness (Jatoi et al., 2006). Similar results were reported on NLP’s high positive direct effect, number of shoots, and rhizome thickness on RYP (Ravi et al., 2017; Islam et al., 2008; Jatoi et al., 2006).

Based on the results of the path coefficient analysis determined for EOY and RYP, it can be concluded that the characters IRW, WMR, and WSR were the most important and could be used for making effective selection program for high rhizome and essential oil yield of ginger.

## Conclusions

Ginger is an important cash crop among all the spices. The extensive use of ginger includes both fresh and dried forms, in candied form, and as an important raw material for various pharmaceutical applications. As ginger is a vegetatively propagated crop, the scope of variability creation ceases. Hence, the proper evaluation of the available genetic baseline of ginger is of utmost importance for selecting and identifying the elite germplasm of ginger. Northeast India has a rich biodiversity, a powerhouse of such wide variability available in nature. In this regard, the detailed evaluation of ginger germplasm across Northeast India could be a high-potential region for selecting ginger germplasm with elite traits. In view of the above reason, the present study was undertaken to assess the morphological diversity of ginger germplasm across Northeast India. The analysis of variance revealed that phenotypic variance is more compared to the genotypic variance, which indicated the influence of environmental impact on ginger germplasm. High heritability coupled with high GA was observed for EOY, followed by RYP. Moderate heritability with moderate GA was observed for LL, NTP, PH, and DMR. Therefore, these characters might be exhibiting additive gene effect predominance. Hence, selecting these traits would be effective for the genetic improvement of RYP and EOY in ginger. Based on the path coefficient analysis results determined for EOY and RYP, it can be concluded that the characters IRW, WMR, and WSR were the most important and appeared promising in improving the overall yield potential of ginger rhizome and essential oil yield.

##  Supplemental Information

10.7717/peerj.15966/supp-1 Supplemental Information 1 Pooled raw data.

## Additional Information and Declarations

### Data Deposition

The following information was supplied regarding data availability: The raw data are available in the Supplemental File.
